# Association of BRAF Mutation Status with Histopathological Characteristics and Survival Outcomes in Stage II–III Malignant Melanoma

**DOI:** 10.3390/ijms27146150

**Published:** 2026-07-09

**Authors:** Vlad Alexandru Gâta, Daniel Corneliu Leucuța, Radu Alexandru Ilieș, Ștefan Țîțu, Ana Maria Mureșan-Bădescu, Delia Nicoară, Ioan Constantin Pop, Alex Victor Orădan, Maximilian Vlad Muntean, Anda Gâta

**Affiliations:** 1 Department of Oncological Surgery and Gynecological Oncology, “Iuliu Hațieganu” University of Medicine and Pharmacy, 400012 Cluj-Napoca, Romania; vlad.gata@umfcluj.ro; 2 Department of Surgical Oncology, “Prof. Dr. Ion Chiricuță” Institute of Oncology, 400015 Cluj-Napoca, Romania; stefan.titu@umfcluj.ro; 3 Department of Medical Informatics and Biostatistics, “Iuliu Hațieganu” University of Medicine and Pharmacy, 400349 Cluj-Napoca, Romania; 4 Faculty of Medicine, “Iuliu Hațieganu” University of Medicine and Pharmacy, 400012 Cluj-Napoca, Romania; ilies.radu.alexandru@elearn.umfcluj.ro (R.A.I.); muresan.badescu.ana.mar@elearn.umfcluj.ro (A.M.M.-B.); 5 Department of Quality Management, “Prof. Dr. Ion Chiricuță” Institute of Oncology, 400015 Cluj-Napoca, Romania; drdelianicoara@gmail.com; 6 Department of Plastic Surgery, “Prof. Dr. Ion Chiricuță” Institute of Oncology, 400015 Cluj-Napoca, Romania; drp.ionut@gmail.com (I.C.P.); alex.oradan@gmail.com (A.V.O.); 7 Department of Plastic and Reconstructive Surgery, “Iuliu Hațieganu” University of Medicine and Pharmacy, 400012 Cluj-Napoca, Romania; 8 Department of Otorhinolaryngology, “Iuliu Hațieganu” University of Medicine and Pharmacy, 400012 Cluj-Napoca, Romania; andapstl@yahoo.com

**Keywords:** BRAF mutation, histopathology, malignant melanoma, prognosis, tumor infiltrating lymphocytes

## Abstract

In patients with advanced or metastatic melanoma, BRAF mutation assessment is routinely performed to identify patients who may benefit from BRAF-targeted therapy. This study aimed to assess the role of BRAF mutation status in relation to histopathological characteristics and survival of patients with stage II and III malignant melanoma. A prospective cohort of 108 patients with pT3 malignant melanoma who were treated in a comprehensive cancer center were included in the analysis. All patients were treated according to contemporary melanoma management guidelines between 2016 and 2024, with a minimum follow-up of 12 months extending to 2025. Overall survival (OS) and progression-free survival (PFS) analyses were performed in the study cohort. The study included 108 patients with stage II–III malignant melanoma, with a mean age of 56.73 ± 13.51 years. Superficial spreading melanoma was the most frequent histological subtype, followed by nodular and acral melanoma. Most tumors were classified as Clark level IV, with a median Breslow thickness of 3 mm, and ulceration was present in the majority of cases. Lymph node involvement was observed in over half of the patients, and BRAF mutations were identified in 56.48% of cases (the most common variant was V600E). Brisk tumor-infiltrating lymphocytes were significantly more frequent in BRAF wild-type tumors compared with BRAF-mutant tumors. When assessing associations with survival, BRAF mutation status was not found to be an independent predictor. In the multivariate Cox model, TIL status was associated with improved OS (HR 3.33, 95% CI 1.41–7.88, *p* = 0.006). In addition, in the multivariate analysis, TIL status was also associated with improved PFS (HR 5.23, 95% CI 2.22–12.3, *p* < 0.001). BRAF wild-type tumors were significantly more likely to exhibit a brisk infiltrate. BRAF mutation status was not found to be an independent predictor of survival. TIL status remained significantly associated with OS and PFS in multivariable analysis.

## 1. Introduction

Malignant melanoma, the cancer of melanocytes, represents the most aggressive and fatal type of skin cancer, with an incidence that has continued to rise in the past 30 years. Even though it usually affects patients in their fifth and sixth decades of life, it represents one of the most common cancers among young adults [1,2], with an estimation of more than 100,000 newly diagnosed patients in 2020 [3,4,5].

The management is complex due to the heterogeneity and the unpredictable behavior of the disease. It involves surgery for stage I and II melanoma patients, with a good prognosis, while for patients with advanced locoregional disease or metastatic disease, outcomes are significantly worse compared to early-stage melanoma. According to SEER data, the 5-year relative survival is nearly 100% when the disease is localized, approximately 76% with regional lymph node involvement, and around 35% for distant metastases, although a subset of patients with nodal disease may still be cured with surgery alone [5]. Although public health campaigns in the past decade have aimed to facilitate early diagnosis, the major improvement in survival for metastatic melanoma has been achieved through modern therapies, including immune checkpoint inhibitors (anti-PD1, anti-CTLA4) and BRAF/MEK targeted therapy, which have significantly changed the prognosis of these patients [6,7,8].

Malignant tumors continue to represent a significant threat to global human health, with mortality being related to metastatic visceral spread. Malignant melanoma, a tumor with a high potential for metastasis, both by hematogenous and lymphatic pathways, accounts for approximately 60% of the deaths caused by skin cancer [9,10,11]. Therefore, the physiopathology of this disease has been intensively studied in the past 20 years; according to the AJCC 8th edition (2017), tumoral progression is linked to a series of complex interactions between the neoplasm and the host’s immune response [12]. In addition, melanoma is considered a highly immunogenic tumor, quickly stimulating immune reactions in the host’s organism [13,14,15] and harboring different genetic alterations in three prominent oncogenes: BRAF, N-RAS, and c-Kit [16,17].

Recent studies involving whole-genome sequencing analyzed different mutation frequencies across several cancers, identifying melanoma with the highest mutational load of all malignant tumors [18,19]. Frequently, the mutations occur within the mitogen-activated protein kinase (MAPK) pathway, which is a key regulator involved in cell growth, proliferation, and survival, via different mechanisms in which the activation of BRAF and N-Ras oncogenes takes place [20,21]. N-RAS and BRAF are two prominent melanoma oncogenes identified in 1982 and 2002. While N-Ras was the first oncogene identified in 15–20% of cutaneous melanomas, the BRAF oncogene was identified later and occurred in 40–60% of cutaneous melanomas [22,23]. BRAF mutations are in more than 90% of cases in codon 600, and the most common BRAF mutation is V600E, in which amino acid valine is substituted with glutamic acid (80%); other BRAF mutations are V600K, V600R, V600D, and in a small proportion, non–V600 mutations (<1%). Notably, the V600K variant has been associated with a worse prognosis under adjuvant BRAF inhibitor therapy and displays distinct biological features compared with the more common V600E [24,25]. These oncogene mutations might occur early in the pathogenesis of melanoma and facilitate tumor progression through uncontrolled cell proliferation [26]. Thus, it is considered that patients with BRAF-mutant melanomas display different clinical features, with a more aggressive behavior occurring in younger patients, with more frequent superficial spreading subtype histology, and a higher tendency to metastasize [24].

BRAF mutation status represents a biomarker used to guide therapy in advanced or metastatic melanoma, where patients are typically treated with BRAF/MEK inhibitor combinations or with immunotherapy, and in some cases, the two approaches are used sequentially [27,28]. Hence, according to the current guidelines, in patients with advanced or metastatic melanoma, assessment of BRAF mutation is routinely done to identify patients that might benefit from anti-BRAF therapy, even though a small proportion will almost develop treatment resistance over time [29].

The importance of understanding the relation between BRAF mutational status and the immune microenvironment is the possibility of refining prognostic assessment in patients with intermediate-risk melanomas (such as pT3), where clinical decision-making in terms of surveillance and systemic therapy remains challenging. Identification of reliable histopathological or molecular biomarkers could therefore provide additional value in stratifying prognosis and guiding therapy.

Although BRAF mutation testing is commonly used to guide systemic therapy in advanced melanoma, its prognostic significance in stage II–III disease is incompletely understood. Particularly, the interaction between BRAF mutational status and the tumor immune microenvironment has not been investigated enough in homogeneous pT3 melanoma cohorts. This study aimed to assess the role of BRAF mutation status in relation with histopathological characteristics and survival of patients with stage II and III malignant melanoma.

## 2. Results

### 2.1. Baseline Clinicopathological Features

The cohort consisted of 108 patients with pT3 melanoma (corresponding to AJCC stage II–III depending on nodal status), aged 56.73 ± 13.51 years (Table 1).

In terms of histopathological characteristics (Table 2), the most common subtype was superficial spreading melanoma (66.67%), followed by nodular (25.93%) and acral melanoma (7.41%). Tumors were predominantly advanced locally, with a median Breslow thickness of 3 mm (IQR 2.5–3.6) and most of the cases were classified as Clark level IV (64.81%). Ulceration was present in 65.74% of patients. Perineural and angiolymphatic invasion were identified in 6.48% and 8.33% of cases, respectively, while regression was observed in 47.22%. Brisk tumor-infiltrating lymphocytes (TILs) were present in 29.63% of cases, and microsatellitosis was found in 16.67%. The median mitotic rate was 8.5 (IQR 3.75–14).

Regarding staging (Table 3), 35.19% of tumors were classified as pT3a, and lymph node involvement occurred in 60.19% of patients (pN1–3). The median number of examined lymph nodes was 4 (IQR 2–13.25), with a median of 1 positive lymph node (IQR 0–2).

BRAF mutations were identified in 56.48% of patients. The most common mutation was V600E (46.3%), followed by V600K (5.56%), while other variants were rare (Table 4).

The primary tumor was most located on the thorax (37.96%), followed by the lower limb (26.85%) and abdomen (13.89%). The single vulvar melanoma case represented a cutaneous melanoma arising in vulvar skin.

Regarding treatment, systemic therapies were administered according to standard clinical indications during the study period. Immune checkpoint inhibitors therapy and BRAF-targeted therapy were used depending on mutation status and disease stage, either in the adjuvant setting or at the time of recurrence (Table 5).

A total of 51 patients (47.22%) developed disease recurrence, including both local and distant relapse. Distant metastases were noticed in 42 patients (38.89%). The distribution of metastatic spread showed a clear predominance of visceral and central nervous system involvement (Table 6). The most frequently affected site was the brain (18.52%), followed by the lungs (13.89%) and regional lymph nodes (7.41%). Less common metastatic locations included the bone (4.63%), liver (3.70%), and peritoneum (1.85%), while rare involvement was observed in the colon (0.93%), mesentery (0.93%), and adrenal glands (0.93%).

When stratified by BRAF mutation status, no statistically significant differences were noticed in the majority of clinicopathological features (Table 7) (*p* > 0.05). However, brisk TILs were significantly more frequent in BRAF wild-type tumors in comparison with BRAF-mutant tumors (42.55% vs. 19.67%, *p* = 0.01).

Moreover, first-line treatment distribution differed significantly between groups (*p* < 0.001) in the univariate model, with BRAF-mutant patients more frequently receiving targeted therapy, while BRAF wild-type patients were more commonly treated with immunotherapy.

### 2.2. Survival Analysis: Overall Survival

**Figure 1:**
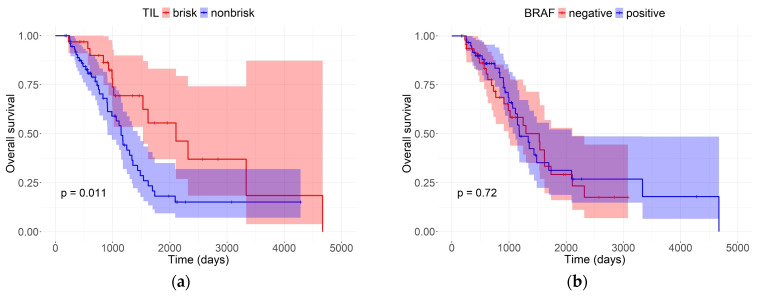
Kaplan–Meier survival analyses for overall survival (OS) stratified by key clinicopathological variables. (**a**) OS according to TIL status, comparing brisk versus non-brisk infiltration; (**b**) OS differences according to BRAF mutation status (mutated versus wild-type). Comparisons were performed using the log-rank test.

**Figure 2:**
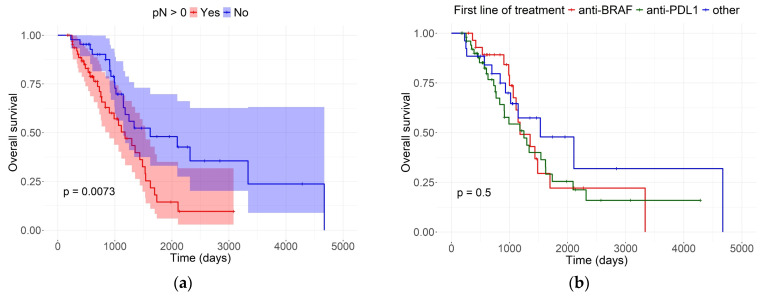
Kaplan–Meier survival analyses for overall survival (OS) stratified by key clinicopathological variables in patients with melanoma. (**a**) OS according to pN status (>0 vs. 0); (**b**) Kaplan–Meier survival analyses for OS stratified by first line of treatment. Comparisons were performed using the log-rank test.

Kaplan–Meier survival analysis was used to evaluate OS according to clinicopathological variables: TIL status, BRAF mutation status, pN category, lymph node involvement, and first-line treatment (Figure 1 and Figure 2).

In the first multivariate Cox regression model (including age, ulceration, TIL status, and BRAF mutation status), TIL remained significantly associated with OS (HR 2.62, 95% CI 1.32–5.21, *p* = 0.006), whereas age greater than 60 years, ulceration, and BRAF mutation status were not statistically significant (Table 8).

In the second multivariate model, additionally adjusted for first-line treatment, TIL status remained significantly associated with OS (HR 3.33, 95% CI 1.41–7.88, *p* = 0.006). Age greater than 60 years, ulceration, BRAF mutation status, and first-line treatment were not statistically significant in the adjusted model.

### 2.3. Survival Analysis: Progression-Free Survival

**Figure 3:**
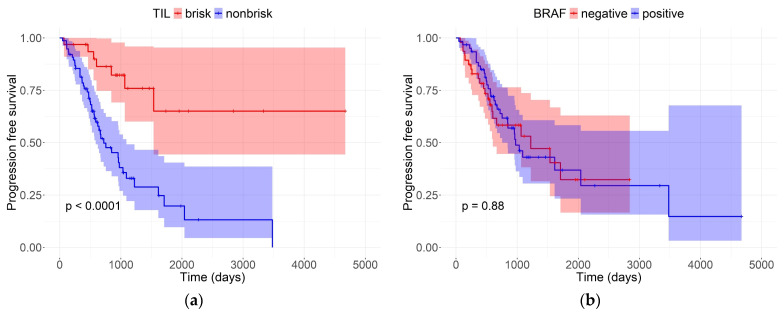
Kaplan–Meier survival analyses for progression-free survival (PFS) stratified by key clinicopathological variables. (**a**) PFS according to TIL status, comparing brisk versus non-brisk infiltration; (**b**) PFS differences according to BRAF mutation status (mutated versus wild-type). Comparisons were performed using the log-rank test.

**Figure 4:**
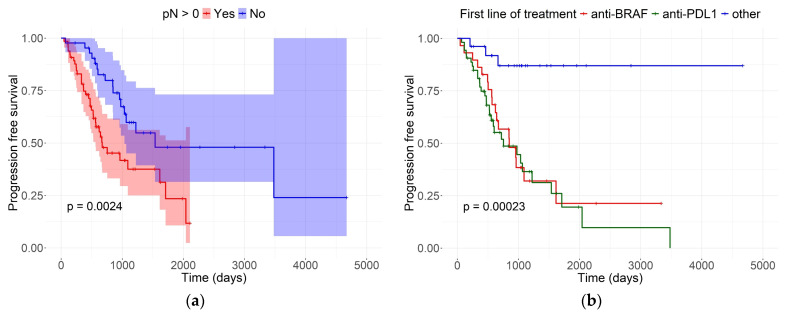
Kaplan–Meier survival analyses for progression-free survival (PFS) stratified by key clinicopathological variables. (**a**) PFS according to pN status (>0 vs. 0); (**b**) Kaplan–Meier survival analyses for PFS stratified by first line of treatment. Comparisons were performed using the log-rank test.

Kaplan–Meier analysis for PFS was performed according to the following clinicopathological variables: TIL status, BRAF mutation status, pN category, lymph node involvement, and first-line treatment (Figure 3 and Figure 4).

In the multivariate Cox regression model (including age, ulceration, TIL status, and BRAF mutation status), TIL remained significantly associated with PFS (HR 5.23, 95% CI 2.22–12.3, *p* < 0.001). Age ≥ 60 years (HR 1.15, 95% CI 0.64–2.06, *p* = 0.65), ulceration (HR 0.99, 95% CI 0.53–1.83, *p* = 0.969), and BRAF mutation status (HR 0.67, 95% CI 0.38–1.20, *p* = 0.178) were not statistically significant in the adjusted model (Table 9).

## 3. Discussion

### 3.1. Summary and Interpretation of the Main Findings

In this cohort of patients with pT3 stage II–III malignant melanoma, BRAF mutations were identified in more than half of the cases, predominantly V600E. Most clinicopathological characteristics did not significantly differ according to BRAF status. However, brisk TIL infiltration was significantly more frequent in BRAF wild-type tumors. While BRAF mutation status was not independently associated with OS or PFS, TIL status was associated with improved OS and PFS in multivariable Cox models.

Additionally, the results show a consistently high-risk population with a strong burden of adverse histopathological and clinical features. The predominance of increased Breslow thickness, ulceration, and frequent lymph node involvement indicates that the majority of patients present with biologically advanced disease, even within the same stage category. This supports the fact that staging alone does not fully capture the biological aggressiveness of melanoma in the current clinical practice.

The high rates of mitotic activity, microsatellitosis, and lymphovascular or perineural invasion further reinforce the aggressive tumor behavior observed in our series. These parameters, altogether, explain the substantial proportion of patients who developed recurrence and distant metastases during follow-up, showing that the natural history of stage II–III melanoma is unfavorable in a significant subset of cases.

Regarding metastatic patterns, the predominance of brain and lung involvement in the cohort reflects the known tropism of melanoma for the central nervous system and visceral organs. This finding is clinically relevant, as it underscores the importance of systematic surveillance strategies, particularly neuroimaging in high-risk patients.

From a molecular standpoint, the BRAF mutation rate was over 50%. The predominance of the V600E variant confirms its central role as the main actionable driver mutation in this population. The stratification of first-line therapy according to BRAF status reflects the integration of molecular diagnostics into clinical decision-making in routine practice, with targeted therapy being preferentially used in mutated cases and immunotherapy in wild-type tumors.

The significant association between BRAF status and TILs represents one of the most relevant biological observations in our dataset. The higher prevalence of brisk TILs in BRAF wild-type tumors indicates a more immunogenic tumor microenvironment in this subgroup, which may partly explain their greater reliance on immunotherapy. The higher prevalence of brisk TIL infiltration in BRAF wild-type melanomas may suggest differences in the tumor immune microenvironment according to BRAF mutational status. However, the biological basis of this association remains unclear and should be interpreted cautiously given the limited cohort size.

This study identified TIL status as the most representative prognostic factor in both PFS and OS analyses. In multivariate analyses, non-brisk TIL was independently associated with worse outcomes, highlighting a relevant role of the host immune response in disease control in melanoma. However, these results should be interpreted with caution, as the multivariable models were not adjusted for nodal status or AJCC stage due to limitations related to the number of events and the risk of overfitting.

In contrast, BRAF mutation status did not show a statistically significant association with either PFS or OS in the multivariate models. Similarly, ulceration and age were not independently associated with survival outcomes after adjustment for other clinicopathological variables.

First-line treatment category, including targeted therapy and immunotherapy-based approaches, did not show an independent effect on survival outcomes in the adjusted analysis, even though differences were observed in univariate comparisons.

Our findings support the biological heterogeneity of stage II–III melanoma, driven by interactions between histopathological, molecular, and immune microenvironment features. These factors not only influence disease progression and metastatic behavior, but they also determine therapeutic strategy. Moreover, the findings emphasize the predominant prognostic relevance of tumor immune microenvironment over classical clinicopathological factors and treatment stratification in this cohort.

### 3.2. Comparison with Existing Literature

The BRAF oncogene represents a serine-threonine kinase down the MAPK pathway, being the most common genetic alteration in melanoma and occurring in 40–60% of patients [17,24,30]. In our study, BRAF mutations were identified in 56.48% of cases, which is in the 40–60% reported range. Also, the most frequent BRAF V600 mutation is V600E [17,24,25], which concurs with our findings, as it was identified in 46.3% of cases. Moreover, it is considered that BRAF-mutated melanomas exhibit different clinical features and correlate with a worse prognosis, with a superficial spreading or nodular melanoma histological subtype while occurring in skin sites that are intermittently exposed to UV radiation [31,32].

In our study, the most common histological subtype that presented BRAF mutation was superficial spreading melanoma, and the median age of BRAF-mutant patients was 56.73 years old.

Also, in the current study, the most common skin site was on the thorax, lower limb, and abdomen (intermittent sun-exposed areas), with a cumulative frequency of 78.7%. These findings align with the statistics presented in published literature [31,32]. In addition, BRAF-mutated melanomas tend to give brain metastases more frequently and are linked with a shorter OS when diagnosed in stage IV, compared to non-BRAF melanomas [24,33,34]. Cerebral metastases were present in 20 patients (18.52%). Although not a primary endpoint of this study, brain involvement is a clinically relevant pattern of metastatic spread in melanoma and was therefore documented within the cohort.

Ellerhurst et al., in a study on 238 primary melanomas, observed that patients with BRAF-mutated tumors presented with a higher tumor stage, even though OS did not differ, concluding that BRAF-mutated melanomas exhibit distinct clinical phenotypes and are associated with a more aggressive behavior [35]. Our findings exhibit a similar pattern regarding the BRAF mutation not influencing the OS and PFS for our cohort of patients.

Moreover, Moreau et al., in a study on 105 stage III cutaneous melanoma, observed that BRAF-mutant patients had a higher risk of death and a shorter distant metastasis-free survival, as well as a higher rate of positive lymph nodes, concluding that BRAF status represents a poor prognostic factor [36]. In addition, Picard et al. studied the prognostic value of BRAF mutation in patients with positive sentinel lymph nodes and observed that even if there was no difference in OS, it is an indicator of poor prognosis for this type of patient [37]. However, controversy exists, as numerous studies validated BRAF mutation as a positive prognosis factor. Therefore, in a study including 93 patients with stage I-III melanoma, the authors concluded that BRAF mutation is associated with a lower risk of developing relapse and distant metastasis, having no prognostic value on overall survival [38]. Moreover, the same conclusion was validated in an analysis of 437 patients with primary melanoma, as no survival differences were found for BRAF-mutant patients [39]. Also, a German study analyzed BRAF and NRAS mutations in melanoma patients. It concluded that NRAS mutant tumors tend to behave more aggressively, whereas BRAF mutant tumors correlate with improved OS [40].

### 3.3. Tumor-Infiltrating Lymphocytes (TILs) as Prognostic Biomarkers

Malignant melanoma represents a highly immunogenic tumor in which the value of immune response plays a vital role in the evolution of the disease and the treatment response rate. Therefore, Clark et al. (1989) [41] elaborated a density grading schema to quantify the value of the immune response, respectively, to assess TIL density and divided the infiltrate into three groups: brisk, non-brisk, and absent. A brisk infiltrate was defined as lymphocytes that are present and infiltrate the entire base of the vertical growth phase of the tumor (peripheral) or show diffuse permeation of the vertical growth phase, whereas a non-brisk infiltrate was defined as a focal TILs infiltrate; absent was considered as an absence of TILs, or if they are present, they were not opposed to malignant cells. Subsequently, a study validated this definition and concluded that when TILs are categorized, they have a substantial predictive value for primary cutaneous melanomas [42]. Furthermore, numerous studies have been conducted to investigate the value of TILs as an essential and independent prognostic factor for patients with malignant melanoma.

Studies conducted by Mandalà et al. on 1251 patients and by Azimi et al. on 1865 patients observed that pronounced lymphocytic infiltrate correlates with improved overall survival [43,44]. Moreover, other studies showed that the absence of TILs may predict an SLN metastasis, confirming that TILs correlate with better patient outcomes [45,46,47]. More recently, a potential association between BRAF and NRAS mutations and TILs was investigated, and a study on 912 patients concluded that only NRAS primary melanomas are independently associated with a lower TILs grade, which may suggest that these melanomas have an immunosuppressed microenvironment [48]. Another study checked for associations of BRAF and NRAS with various clinicopathological characteristics and observed that BRAF-mutant melanomas were associated with a moderate to pronounced infiltrate, whereas NRAS mutations were correlated with minimal or absent infiltrate; the authors concluded that a BRAF mutation may elicit a more robust immunological response compared to NRAS or BRAF wild-type melanomas [49]. These differences, when compared to previous larger studies [48,49], may be related to the characteristics of our cohort (single-center, relatively small, and restricted exclusively to pT3 melanomas surgically treated in our institution). However, our study does not provide a clear biological explanation for this divergence. The findings should therefore be regarded as hypothesis-generating and interpreted with caution until validated in larger, heterogeneous series.

Starting from the previous conclusions, Leslie et al. assessed whether FOXP3 T regulatory lymphocytes are associated with BRAF mutation and response to BRAF inhibitor therapy [50]. T regulatory cells are a subpopulation of lymphocytes that exhibit an immunosuppressive effect, facilitating tumor proliferation and expressing the transcription factor Forkhead Box p3 (FOXP3); an increased density of these cells is correlated with a poor prognosis, not only in melanoma but also in other cancers, such as hepatocellular, gastric, and cervical cancer.

Moreover, high circulating Treg levels and intratumoral FOXP3 lymphocytes are associated with melanoma progression and a higher frequency of SLNB positivity [51]. Therefore, Leslie et al. reported an association between BRAF mutant melanomas and a high density of intratumoral FOXP3 Tregs, which may suggest tumor progression through the lack of antitumor immune response. In addition, in the same study, FOXP3 Treg density could not be assessed as a predictive factor for the response to BRAF inhibitor therapy [50].

In a previous study conducted by Gâta et al., the prognostic role of Forkhead Box P3 expression in malignant melanoma was evaluated in a cohort of 79 patients with pT3 tumors, demonstrating a high prevalence of FoxP3 positivity (81%) and a tumor relapse rate of 36.7%. FoxP3 expression was significantly associated with worse outcomes, including an increased risk of relapse (37%, *p* = 0.007), higher mortality (HR ≈ 12.35, *p* = 0.004), and a higher rate of lymph node metastasis (~50% vs. <7%, *p* = 0.03). Survival analysis further showed reduced OS in FoxP3-positive patients (5-year OS ~30.5%, *p* < 0.001), while multivariate analysis confirmed FoxP3 expression (HR = 12.77, *p* = 0.014) and nodal involvement (HR = 1.81, *p* = 0.003) as independent predictors of mortality [52]. These findings emphasize that increased infiltration of FoxP3-positive regulatory T cells reflects a strongly immunosuppressive tumor microenvironment, which might promote tumor progression and immune evasion, hence supporting its role as a clinically relevant prognostic biomarker in melanoma.

### 3.4. Limitations of the Current Study

Our study has limitations that are in line with the existing studies in the literature. Causal relationships cannot be inferred. Confounding is a known issue of observational studies. Although we used multivariate models to control for known confounders, residual confounding remains. Even though we included a small cohort of patients, this study is one of the few studies in the literature that evaluated a potential association between BRAF-mutated pT3 melanomas and various clinicopathological features, as we consider that a pT3 stage may represent a borderline stage in the treatment of melanoma, with an unpredictable prognosis and evolution of the disease.

Even if the strict inclusion criteria contributed to the homogeneity of the cohort and reduced variability related to staging and treatment protocols, they might also limit the generalizability of the findings to the broader melanoma population. The current study included exclusively pT3 melanomas that were treated in a single tertiary cancer center; therefore, the results may not fully reflect the biological behavior and prognostic impact of BRAF mutations in thinner melanomas, more advanced primary tumors, or patients managed in different healthcare settings. Extrapolation of these findings should be performed with caution and further multicentric studies including more heterogeneous melanoma populations are warranted.

Another limitation is that the study period overlapped with changes in melanoma staging (AJCC 8th edition [12]) and with the introduction of modern adjuvant systemic therapies, which may limit the comparability with current clinical practice. In this context, adjuvant systemic treatments were heterogeneous during the study interval. Patients received different systemic treatment strategies, including immunotherapy, targeted therapy, chemotherapy or surveillance, according to molecular status and clinical indications. Therefore, treatment-selection bias cannot be excluded, since BRAF-mutant patients were more likely to receive targeted therapy, while BRAF wild-type patients more frequently underwent immunotherapy-based approaches. Although treatment category was included in the multivariate analysis, residual confounding related to therapeutic heterogeneity may still have influenced survival outcomes.

A further limitation is the potential for immortal-time bias related to the timing of systemic therapy administration, as treatments were initiated at different time points (adjuvant or after recurrence), while survival was calculated from the date of surgical excision of the primary tumor.

Melanoma-specific survival or disease-specific survival could not be reliably assessed because of incomplete availability of cause-of-death data across the entire cohort. This was the reason why overall survival was used as the primary survival endpoint, which may be influenced by non–melanoma-related mortality in a long-term cohort.

In addition, the absence of statistically significant survival differences according to BRAF mutational status should not be interpreted as evidence of absence of prognostic effect, particularly given the relatively limited sample size.

Another limitation of this study is that lymph node involvement (despite being strongly associated with survival in univariate analysis) was not included in the final multivariate Cox models to avoid model overfitting given the number of events and variable interdependence. Hence, residual confounding by nodal disease burden cannot be excluded.

We also acknowledge that the distribution of distant recurrence sites in our cohort included atypical localizations for melanoma (e.g., sigmoid colon, adrenal gland). This reflects the real findings from our small cohort and should be interpreted with caution, as unusual metastatic presentations may become more evident in limited series.

Importantly, BRAF V600 wild-type status reflects the absence of detectable V600 mutations using a codon 600–targeted assay, rather than the absence of all possible BRAF alterations.

Finally, the relatively limited sample size may have reduced the statistical power to detect moderate survival differences according to BRAF mutational status.

## 4. Materials and Methods

### 4.1. Study Design and Setting

This was a single-center prospective observational cohort study conducted at the Oncology Institute “Prof. Dr. I. Chiricuță”, Cluj-Napoca, Romania, a tertiary comprehensive cancer center. A consecutively enrolled patient cohort was established based on institutional records. Baseline clinical and pathological data were obtained at the time of inclusion, and patients were subsequently followed longitudinally according to a predefined study protocol for survival outcomes assessment.

Patients were consecutively enrolled between January 2016 and November 2024, with follow-up completed until November 2025. Written informed consent was obtained from all study participants prior to inclusion.

The study protocol was approved by the Ethics Committee of the Oncology Institute “Prof. Dr. I. Chiricuță”, Cluj-Napoca, Romania (approval number 42.1/8 December 2015). Patient enrollment and data collection were initiated only after ethics approval had been obtained.

### 4.2. Participants

Strict eligibility criteria were applied in order to obtain a homogeneous cohort of surgically treated pT3 melanomas with complete molecular profiling and follow-up data.

Inclusion criteria:

(1) histologically confirmed malignant melanoma;
(2) patients who have undergone surgery exclusively at our cancer center;
(3) patients with stage II and III malignant melanoma (pathological T3 staging—Breslow thickness 2.01–4.00 mm, with or without lymph node involvement), according to AJCC 8th edition [12];
(4) patients whose BRAF mutation status was evaluated;
(5) patients with adequate follow-up, defined as a minimum of 12 months from diagnosis, according to the ESMO Clinical Practice Guidelines [29].

Exclusion criteria:

(1) patients who were not fully treated in our cancer center; patients who did not have an evaluation of the regional lymph nodes;
(2) patients who underwent nodal staging more than 8 weeks after the diagnosis.

The restriction to pT3 melanomas reduced stage-related biological heterogeneity and allowed a more focused evaluation of the interaction between BRAF status and immune microenvironment.

Follow-up consisted of regular clinical examinations and imaging investigations at intervals recommended for stage II–III melanoma. Lymph node status was evaluated by surgery, either by direct lymph node dissection (LND) or sentinel lymph node biopsy (SLNB) and completed with lymph node dissection if positive within two months maximum from the initial diagnosis.

To ensure a minimum follow-up duration of 12 months for all included patients, patient enrollment was completed by November 2024, while follow-up data were updated until November 2025.

### 4.3. Data Sources and Measurements

Clinical and histopathological data were prospectively collected and stored in a dedicated institutional database and subsequently analyzed for this study.

Malignant melanoma was staged according to the latest staging system of the American Joint Committee on Cancer (AJCC, 8th edition, 2018) [12].

Systemic treatment allocation was performed according to contemporary European Melanoma Guidelines (ESMO) [29], molecular profile, disease stage, and individual patient clinical characteristics at the time of treatment initiation. Systemic therapies were administered either in the adjuvant setting or after disease recurrence, according to standard clinical practice and multidisciplinary decisions during the study period. For survival analyses, systemic treatment was included as a baseline categorical covariate, defined according to the first systemic therapy received during the disease course (either in the adjuvant setting or at recurrence).

#### 4.3.1. BRAF Mutation Testing

Formalin-fixed, paraffin-embedded tissue blocks of the primary melanomas were retrieved from the Department of Pathology, and BRAF mutation analysis was performed on primary tumor samples. Each block was recut for DNA extraction, and these sections were deparaffinized using xylene and ethanol for purification. Then, using Digestion buffer and Proteinase K, samples were incubated over 24 h at 50 degrees Celsius. After using RNase A, the binding buffer and ethanol are centrifuged, two wash buffers are added, centrifuged again, and then incubated for five more minutes. The concentration is detected using the NanoDrop Spectrophotometer (Thermo Fisher Scientific, Waltham, MA, USA). After DNA extraction is successful, PCR is performed using the BRAF Codon 600 Mutation Analysis kit (EntroGen, Inc., Los Angeles, CA, USA), with 40 cycles of amplification.

BRAF mutation status refers to the detection of BRAF V600 mutations (including V600E, V600K, V600D, V600R and related variants) using the aforementioned PCR-based assay. The assay is designed to detect clinically relevant hotspot mutations in codon 600. Non–V600 BRAF mutations were not assessed and may not be detected by this method. Macrodissection was performed when required to enrich tumor cellularity prior to DNA extraction.

#### 4.3.2. Assessment of TILs

The evaluation of TILs was based on hematoxylin and eosin-stained sections of the primary tumors by an experienced pathologist. The evaluator was blinded to BRAF mutation status and survival outcomes during assessment.

For consistency, tumor-infiltrating lymphocytes (TILs) were initially evaluated in three standard categories: brisk (lymphocytes diffusely infiltrating the tumor or present along the entire base of the vertical growth phase), non-brisk (focal or discontinuous infiltration), and absent (no lymphocytic infiltration opposing tumor cells). For statistical analyses, due to the low frequency of absent cases, non-brisk and absent categories were combined into a single non-brisk group.

### 4.4. Statistical Analysis and Survival Analysis

Qualitative data were described numerically using numbers and percentages, respectively, and represented by column-type graphs. Quantitative data were described by mean and standard deviation, or by median and interquartile range (quartiles 1 and 3). Associations between qualitative variables were evaluated using contingency tables, which included absolute frequencies and percentages per row, represented by mosaic or bar graphs. The existence of an association was tested using the Chi-Square test or Fisher’s exact test. When assessing the relationship between dichotomous qualitative variables, indicators such as odds ratios and attributable risk, with 95% confidence intervals, were utilized. Associations between clinicopathological variables and BRAF mutational status were evaluated using multivariate logistic regression models. For these models, multicollinearity was assessed, and model fit was evaluated using the Hosmer-Lemeshow test. The predictive capacity of the models was assessed by the overall percentage of correct classification and the area under the receiver operating characteristic curve. Results are reported with 95% confidence intervals. The Mann-Whitney U test was used to assess differences between two independent groups of quantitative data, and the differences between groups were quantified by the median difference with associated 95% confidence intervals. Multivariate models were constructed using clinically relevant variables with established prognostic significance in melanoma. To reduce the risk of model overfitting given the number of survival events, the number of variables included in the Cox regression models was intentionally limited. Inclusion of highly interrelated prognostic variables, such as nodal status and AJCC stage, could have resulted in model instability and overfitting. Model selection was based on clinical and histopathological relevance, and no AIC-based stepwise procedure was applied.

Overall survival (OS) was defined as the time from the date of surgical excision of the primary melanoma to death from any cause or last follow-up. Progression-free survival (PFS) was defined as the time from surgery to the first occurrence of local recurrence, in-transit metastasis, regional lymph node recurrence, distant metastasis, or death from any cause. Disease-specific survival (DSS) was not evaluated because of incomplete availability of cause-of-death data. In this study, we used the term PFS for consistency with the study design and previously published literature in similar postoperative melanoma cohorts, although we acknowledge that relapse-free survival (RFS) is more frequently used in the adjuvant setting.

Survival data were described by presenting the number of events, censored data, and the percentage of survival at 5 years, graphically represented by Kaplan-Meier survival curves. Comparisons between groups for survival data were made using the log-rank test. Multivariate Cox proportional hazards regression models were used to evaluate the association between selected clinicopathological variables and survival outcomes, with hazard ratios (HRs) and 95% confidence intervals (CIs) reported. The assumption of proportional hazards was verified graphically using Schoenfeld residuals and a statistical test. A significant threshold of 0.05 was used for all tests, and two-sided *p*-values were considered where applicable. Statistical analyses and graphical representations were performed using R version 4.3.2.

## 5. Conclusions

In our cohort of pT3 melanoma patients the BRAF mutation status was not independently associated with survival outcomes, while TIL status remained a consistent prognostic factor. Brisk TIL infiltration was significantly more frequent in BRAF wild-type melanomas, supporting the potential prognostic relevance of the tumor immune microenvironment in intermediate-thickness melanoma. These findings suggest that the prognostic impact in this cohort may be driven by tumor immune microenvironment rather than BRAF mutational status. While our cohort was single-center and relatively limited in size, its strict homogeneity strengthens the reliability of these observations.

## Figures and Tables

**Table 1:** Baseline demographic, histopathological, and primary tumor characteristics.

Characteristic	Value
Age (years), mean (SD)	56.73 (13.51)
Subtype, *n* (%)	
Acral melanoma	8 (7.41)
Nodular melanoma	28 (25.93)
Superficial spreading melanoma	72 (66.66)
Primary tumor location, *n* (%)	
Thorax	41 (37.96)
Lower limb	29 (26.85)
Abdomen	15 (13.89)
Head and neck	13 (12.04)
Upper limb	9 (8.33)
Vulva	1 (0.93)

SD, standard deviation.

**Table 2:** Histopathological prognostic features.

Characteristic	Value
Clark level, *n* (%)	
II	1 (0.93)
III	32 (29.63)
IV	70 (64.81)
V	5 (4.63)
Ulceration, *n* (%)	71 (65.74)
Perineural invasion, *n* (%)	7 (6.48)
Angiolymphatic invasion, *n* (%)	9 (8.33)
Regression, *n* (%)	51 (47.22)
Brisk TILs, *n* (%)	32 (29.63)
Microsatellitosis, *n* (%)	18 (16.67)

TILs, tumor infiltrating lymphocytes.

**Table 3:** Pathological staging.

Characteristic	Value
pT stage, *n* (%)	
pT3a	38 (35.19)
pT3b	70 (64.81)
pN stage, *n* (%)	
N0	43 (39.81)
N1	42 (38.89)
N2	10 (9.26)
N3	13 (12.04)

**Table 4:** Molecular profile (BRAF status and mutations).

Characteristic	Value
BRAF status, *n* (%)	
Wild-type	47 (43.52)
Mutant	61 (56.48)
BRAF mutation subtype, *n* (%)	
V600E	50 (46.30)
V600K	6 (5.56)
V600D	1 (0.93)
V600M	2 (1.85)
V600R	2 (1.85)

BRAF, RAF murine sarcoma viral oncogene homolog B.

**Table 5:** Systemic treatment modalities in the study cohort.

Characteristic	Value
First-line treatment, *n* (%)	
Anti-BRAF therapy	29 (26.85)
Immune checkpoint inhibitors therapy	53 (49.07)
Chemotherapy	2 (1.85)
Surveillance	24 (22.22)
Treatment regimens administered, *n* (%)	
No systemic treatment/surveillance	24 (22.22)
Dabrafenib + trametinib	25 (23.15)
Dabrafenib + trametinib + nivolumab	2 (1.85)
Dabrafenib + trametinib + nivolumab + ipilimumab	1 (0.93)
Dabrafenib + trametinib + nivolumab + pembrolizumab	1 (0.93)
Dacarbazine	2 (1.85)
Nivolumab	18 (16.67)
Nivolumab + dabrafenib + trametinib	3 (2.78)
Nivolumab + ipilimumab	7 (6.48)
Pembrolizumab	23 (21.30)
Pembrolizumab + ipilimumab	2 (1.85)

BRAF, RAF murine sarcoma viral oncogene homolog B.

**Table 6:** Clinical outcomes and metastatic pattern.

Characteristic	Value
Recurrence (local/distant), *n* (%)	51 (47.22)
Distant metastasis, *n* (%)	42 (38.89)
ITM positive, *n* (%)	11 (10.19)
Metastatic sites, *n* (%)	
Cerebral	20 (18.52)
Pulmonary	15 (13.89)
Lymph node	8 (7.41)
Bone	5 (4.63)
Hepatic	4 (3.70)
Peritoneal	2 (1.85)
Colon	1 (0.93)
Mesentery	1 (0.93)
Adrenal	1 (0.93)

ITM, in transit metastasis.

**Table 7:** Clinicopathological characteristics stratified by BRAF mutation status.

Characteristic	BRAF Wild-Type (*n* = 47)	BRAF-Mutant (*n* = 61)	*p*-Value
Subtype, *n* (%)			0.515
Acral melanoma	4 (8.51)	4 (6.56)	
Nodular melanoma	14 (29.79)	14 (22.95)	
Superficial spreading melanoma	29 (61.70)	43 (70.49)	
Age ≥ 60 years	25 (53.19)	26 (42.62)	0.275
Ulceration	29 (61.70)	42 (68.85)	0.438
Brisk TILs	20 (42.55)	12 (19.67)	0.010
Perineural invasion, *n* (%)	4 (8.51)	3 (4.92)	0.466
Angiolymphatic invasion, *n* (%)	3 (6.38)	6 (9.84)	0.729
Regression, *n* (%)	20 (42.55)	31 (50.82)	0.394
Microsatellitosis, *n* (%)	7 (14.89)	11 (18.03)	0.664
pT3a, *n* (%)	19 (40.43)	19 (31.15)	0.317
Positive lymph nodes, *n* (%)	26 (55.32)	39 (63.93)	0.365
ITM positive, *n* (%)	5 (10.64)	6 (9.84)	1
Distant metastases	17 (36.17)	25 (40.98)	0.611
Cerebral metastases, *n* (%)	9 (19.15)	11 (18.03)	0.882
Pulmonary metastases, *n* (%)	10 (21.28)	5 (8.2)	0.051
Bone metastases, *n* (%)	0 (0)	5 (8.2)	0.067
First-line therapy: anti-BRAF	0 (0)	29 (47.54)	<0.001
First-line therapy: immune checkpoint inhibitors	31 (65.96)	22 (36.07)	<0.001

BRAF, RAF murine sarcoma viral oncogene homolog B; ITM, in-transit metastases; TILs, tumor infiltrating lymphocytes.

**Table 8:** Multivariate Cox proportional hazards analysis for overall survival.

Variable	Adjusted HR	95% CI	*p*-Value
Model 1			
Age ≥ 60 years	1.40	(0.78–2.49)	0.256
Ulceration	1.01	(0.55–1.86)	0.983
Tumor-infiltrating lymphocytes (non-brisk vs. brisk)	2.62	(1.32–5.21)	0.006
BRAF mutation (mutant vs. wild-type)	0.78	(0.44–1.40)	0.409
Model 2 (including treatment)			
Age ≥ 60 years	1.42	(0.80–2.52)	0.228
Ulceration	1.07	(0.57–2.02)	0.828
Tumor-infiltrating lymphocytes (non-brisk vs. brisk)	3.33	(1.41–7.88)	0.006
BRAF mutation (mutant vs. wild-type)	0.76	(0.37–1.56)	0.457
First-line therapy (anti-BRAF vs. other)	0.66	(0.24–1.80)	0.420
First-line therapy (immune checkpoint inhibitors vs. other)	0.64	(0.25–1.63)	0.349

BRAF, RAF murine sarcoma viral oncogene homolog B; HR, hazard ratio; CI, confidence interval.

**Table 9:** Multivariate Cox proportional hazards analysis for progression-free survival.

Variable	Adjusted HR	95% CI	*p*-Value
Age ≥ 60 years	1.15	(0.64–2.06)	0.650
Ulceration	0.99	(0.53–1.83)	0.969
Tumor-infiltrating lymphocytes (non-brisk vs. brisk)	5.23	(2.22–12.30)	<0.001
BRAF mutation (mutant vs. wild-type)	0.67	(0.38–1.20)	0.178

BRAF, RAF murine sarcoma viral oncogene homolog B. HR, hazard ratio; CI, confidence interval.

## Data Availability

Data are available upon reasonable request from the corresponding author.
